# Otologic Axis and Sleep-Disordered Breathing in Achondroplasia: Age-Structured Cohort Findings

**DOI:** 10.3390/healthcare14010006

**Published:** 2025-12-19

**Authors:** Cristina Popescu, Rebecca-Cristiana Serban, Andreea Mituț-Velișcu, Andrei Costache, Raluca-Ioana Teleanu, Diana Ionescu, Cristian Arsenie, Renata-Maria Varut, Ion-Dorin Pluta, Virginia Maria Radulescu, Ioana Streață

**Affiliations:** 1 ENT County Hospital Craiova, Discipline of Anatomy, Department of Anatomy, University of Medicine and Pharmacy, 200349 Craiova, Romania; 2 Regional Centre of Medical Genetics Dolj, Emergency County Hospital Craiova, 200642 Craiova, Romania; 3 Laboratory of Human Genomics, University of Medicine and Pharmacy of Craiova, 200638 Craiova, Romania; 4 Department of Biophysics, University of Medicine and Pharmacy of Craiova, 200638 Craiova, Romania; 5 Department of Neuroscience, “Carol Davila” University of Medicine and Pharmacy, 020021 Bucharest, Romania; raluca.teleanu@umfcd.ro; 6 Department of Pediatric Neurology, “Dr. Victor Gomoiu” Children’s Hospital, 022102 Bucharest, Romania; 7 Department of ENT, “Dr. Victor Gomoiu” Children’s Hospital, 022102 Bucharest, Romania; 8 Faculty of Medicine, Titu Maiorescu University, 022102 Bucharest, Romania; 9 Research Methodology Department, Faculty of Pharmacy, University of Medicine and Pharmacy of Craiova, 200349 Craiova, Romania; 10 Faculty of Medical and Behavioral Sciences, Constantin Brâncuși University of Târgu Jiu, 210135 Târgu Jiu, Romania; 11 Department of Medical Informatics and Biostatistics, Faculty of Medicine, University of Medicine and Pharmacy of Craiova, 200349 Craiova, Romania; virginia.radulescu@umfcv.ro

**Keywords:** achondroplasia, otitis media, obstructive sleep apnea (OSA), Eustachian-tube dysfunction (ETD), pediatric ENT, age-class stratification, absolute risk difference, retrospective cohort

## Abstract

**Background/Objectives**: Achondroplasia is linked to distinctive ear–nose–throat (ENT) morbidity, yet quantitative age-structured profiles and actionable correlates remain incompletely defined. This study mapped ENT phenotypes in a consecutive cohort and examined the achondroplasia subset for prevalence, co-occurrence, age dynamics, and parsimonious risk models. **Methods**: Retrospective observational analysis (1 February 2023–31 January 2025). Narrative “ENT complications” were dictionary-mapped to five non-exclusive categories: otitis media, adenotonsillar/apnea—obstructive sleep apnea (OSA), audiologic/Eustachian-tube dysfunction (ETD), nasopharyngeal/upper-respiratory (URT), and extra-ENT. Proportions used Wilson 95% confidence intervals (CIs). Pairwise associations used Fisher’s exact tests with Benjamini–Hochberg false discovery rate (BH-FDR). Age was summarized by a four-level age-class schema (AC-4: 0–2, 3–5, 6–12, ≥13 years) and a two-level sensitivity contrast (AC-2: ≤5 vs. >5 years). **Results**: Of 83 patients, 64 (77.1%) had achondroplasia. In achondroplasia, otitis media occurred in 51.6% and OSA in 28.1%; versus non-achondroplasia, ARDs were +35.8 and +28.1 percentage points (BH-FDR adjusted). Within achondroplasia, otitis media co-occurred with OSA (odds ratio [OR] 4.97; q = 0.012) and with ETD (OR 7.25; q = 0.012). OSA increased across AC-4 to school age (*p*-trend = 0.0548). In parsimonious models, otitis media independently predicted ETD and OSA. A five-item ENT-burden score discriminated otologic and adeno-tonsillar interventions (AUC 0.83–0.93). **Conclusions**: Achondroplasia shows a concentrated ENT burden dominated by otitis media and OSA, with large adjusted absolute differences versus non-achondroplasia. Otitis media functions as a practical clinical marker for both OSA and ETD, while a compact burden score may assist intervention triage.

## 1. Introduction

Achondroplasia is the most frequent skeletal dysplasia and the most common genetic cause of short stature worldwide. It results from a pathogenic gain-of-function variant in the fibroblast growth factor receptor 3 (FGFR3) gene that leads to abnormal endochondral ossification and disproportionate limb shortening [1]. The typical phenotype comprises rhizomelic shortening of the limbs, macrocephaly, frontal bossing, and midface hypoplasia, but the consequences of this monogenic disturbance extend well beyond the skeleton. Over recent decades, multidisciplinary studies have demonstrated that achondroplasia is a multisystem condition affecting neurological, respiratory, otolaryngologic, and cardiovascular domains throughout the lifespan [1,2].

Among these comorbidities, disorders of the ear, nose, and throat (ENT) represent an especially important clinical burden. Children with achondroplasia frequently develop early otologic disease, upper airway obstruction, and sleep-disordered breathing. These complications arise from the unique craniofacial morphology that characterizes the disorder—short cranial base, midface hypoplasia, and narrow nasopharyngeal space—which together alter ventilation of the middle ear and upper airway [3]. The resulting mechanical and functional impairments predispose to chronic otitis media, recurrent effusions, Eustachian-tube dysfunction, and progressive hearing loss.

Otitis media with effusion (OME) and recurrent acute otitis media are common findings in infancy and early childhood, but they occur with striking frequency and persistence in achondroplasia. Collins and Choi reviewed otolaryngologic manifestations in a paediatric cohort and found that more than two-thirds of children exhibited middle-ear effusion or recurrent infection and that half required tympanostomy tube insertion [3]. Subsequent studies have confirmed a high prevalence of conductive hearing loss secondary to middle-ear pathology, suggesting that chronic inflammation and structural crowding in the nasopharynx play central roles [2,4]. Hearing impairment has long-term developmental consequences, including delayed speech acquisition and impaired social communication, highlighting the importance of early detection and intervention.

Anatomical and functional mechanisms underlying these otologic findings have been extensively discussed. Midface hypoplasia shortens the distance between the nasopharynx and tympanic cavity, leading to a more horizontal Eustachian tube and ineffective clearance of middle-ear secretions. The reduced size of the skull base and narrowed upper airway further limit mucosal airflow and drainage, creating conditions conducive to recurrent effusion. Moreover, hypertrophy of adenoidal and tonsillar tissue—a frequent occurrence in childhood—exacerbates obstruction of the Eustachian tube orifice. Collectively, these factors generate a self-reinforcing cycle of impaired ventilation, effusion, and infection [3,4].

In parallel with otologic morbidity, respiratory compromise and sleep-disordered breathing constitute major sources of morbidity in achondroplasia. Obstructive sleep apnea (OSA) is increasingly recognised as a core component of the phenotype. Craniofacial restriction, macroglossia, adenotonsillar hypertrophy, and small airway calibre predispose to partial or complete collapse during sleep [5]. The consequences extend beyond sleep fragmentation and daytime fatigue to include neurocognitive deficits, cardiovascular stress, and metabolic dysregulation. In paediatric cohorts, polysomnographic evaluation has revealed abnormal respiratory indices in a large majority of patients, with some demonstrating severe oxygen desaturation and hypercapnia during sleep [5,6].

The interplay between otologic disease and sleep-disordered breathing is particularly relevant. Middle-ear dysfunction and chronic nasal obstruction can promote mouth-breathing, altered craniofacial growth, and further upper airway collapse, while repeated nocturnal hypoxia and inflammation may, in turn, influence mucosal and Eustachian-tube function. Although the causal direction of this relationship remains incompletely understood, clinical evidence suggests that these conditions often coexist and may reinforce each other’s severity [3,5]. Understanding their co-occurrence provides an opportunity for earlier identification of at-risk children and targeted intervention.

Management guidelines now emphasise early and systematic ENT and sleep evaluation as integral components of lifelong care for individuals with achondroplasia. The International Consensus Statement published in Nature Reviews Endocrinology in 2022 recommends proactive surveillance beginning in infancy, with periodic audiologic assessment, tympanometry, and polysomnography when symptoms or risk factors are present [1]. Similarly, the European Achondroplasia Forum has proposed practical guidance for the assessment and treatment of sleep-disordered breathing, stressing the need for coordinated management among geneticists, pulmonologists, and otolaryngologists [5]. The consensus highlights that adenotonsillectomy, tympanostomy, and continuous positive airway pressure (CPAP) therapy are often required sequentially or in combination to address the multifactorial pathophysiology of upper airway obstruction in this population.

Despite these advances, critical knowledge gaps persist. The majority of published studies remain retrospective or cross-sectional, and sample sizes are frequently small due to the rarity of achondroplasia. Consequently, quantitative data describing the prevalence, severity, and age-related progression of ENT disorders remain scarce. The co-occurrence and potential bidirectional influence of otitis media, Eustachian-tube dysfunction, and OSA have not been systematically evaluated in well-defined cohorts. Furthermore, many existing reports originate from tertiary centres, introducing potential referral bias. These limitations hinder the development of predictive models and evidence-based algorithms for screening and follow-up.

Another unresolved issue is how these complications evolve over time and how early intervention might alter long-term outcomes. In the general population, persistent otitis media with effusion tends to resolve spontaneously in most cases, whereas in achondroplasia it may persist well into adolescence and adulthood [2,3]. Similarly, while adenotonsillectomy can substantially reduce OSA severity in otherwise healthy children, relapse or residual obstruction is frequent in achondroplasia due to the fixed skeletal component of the airway restriction [5,6]. Understanding age-dependent patterns is therefore essential for designing surveillance schedules that are both effective and resource-efficient.

In recent years, new therapeutic possibilities such as vosoritide—an analogue of C-type natriuretic peptide that counteracts FGFR3 overactivation—have shown efficacy in promoting linear growth in achondroplasia [7]. Although primarily developed to address skeletal growth deficits, there is growing interest in whether improved craniofacial development through long-term therapy might also influence ENT outcomes. To explore such secondary benefits, reliable baseline data describing the natural history of ENT morbidity are indispensable.

The present study addresses these knowledge gaps by providing a quantitative mapping of ENT phenotypes in a consecutive cohort of patients evaluated in a paediatric setting, with specific attention to achondroplasia. By analysing prevalence, co-occurrence, and age dynamics of otitis media, Eustachian-tube dysfunction, and obstructive sleep apnea, we aim to characterise the internal structure of ENT morbidity and to identify clinically actionable correlates. The findings are expected to inform parsimonious risk models and practical scoring systems that can guide early referral and intervention in both specialist and community settings.

## 2. Materials and Methods

### 2.1. Study Design and Setting

This retrospective observational analysis included consecutively recorded patients evaluated between 1 February 2023 and 31 January 2025. The unit of analysis was the individual patient. For patients with multiple entries, information was harmonized at the patient level; the variable “Latest presentation year” anchored temporal summaries. All patients with available age and gender and at least one relevant clinical entry during the observation window were eligible. Records missing an essential field for a given analysis were excluded from that analysis only; complete-case denominators are reported in the Results.

ENT evaluations were performed by board-certified otolaryngologists, while routine examinations at initial presentation were occasionally conducted by pediatric generalists before specialist referral. Audiometry and tympanometry were available in the tertiary center for children ≥ 3 years, but utilization varied with age and cooperation. Missingness was non-differential across age bands for core ENT categories; however, severity/type annotations for OSA were sparse because polysomnography was not systematically performed in this retrospective dataset. Consequently, OSA severity labels reflect clinician-reported impressions rather than polysomnography (PSG)-confirmed indices.

To enhance coding consistency in this retrospective design, a structured verification step was implemented. A second investigator independently reviewed a random subset (~15%) of records to confirm concordance in the abstraction of ENT categories and narrative-to-dictionary mappings. Agreement exceeded 90%, and any discrepancies were resolved through consensus. Although full dual re-abstraction was not feasible, this sensitivity check increases confidence in the stability of the coding framework used in this study.

### 2.2. Variable Operationalization

Abbreviations are expanded at first mention and used thereafter. Key terms include the following: Age-Class-4 (AC-4; four-level age-class schema), Age-Class-2 (AC-2; two-level age-class schema), obstructive sleep apnea (OSA), Eustachian-tube dysfunction (ETD), upper-respiratory tract (URT), Benjamini–Hochberg false discovery rate (BH-FDR), events per variable (EPV), area under the receiver operating characteristic curve (AUC), absolute risk difference (ARD), Kolmogorov–Smirnov (KS), and Shapiro–Wilk (SW).

Age was analyzed both continuously and by age classes using two pre-specified schemes. Age-Class-4 (AC-4) comprised four developmental classes—0–2, 3–5, 6–12, and ≥13 years—selected to reflect stages of ear–nose–throat (ENT) risk (Eustachian-tube immaturity and early otitis; preschool adenotonsillar hypertrophy; school-age transition; adolescence) while maintaining adequate cell sizes. Age-Class-2 (AC-2) provided a two-level sensitivity contrast—≤5 vs. >5 years—to test the robustness of age gradients. Unless stated otherwise, “AC-4” and “AC-2” denote these age-class schemas. Continuous age (years) was retained for modeling where appropriate. Age-class boundaries were selected a priori based on developmental phases known to influence ENT risk: (0–2 years) Eustachian-tube immaturity; (3–5 years) peak adenotonsillar hypertrophy; (6–12 years) school-age transition with stabilization of craniofacial growth; and (≥13 years) adolescent airway maturation. Cutoffs were also constrained by events-per-variable considerations to maintain adequate cell sizes.

The principal diagnosis was abstracted as recorded. Categories observed in this cohort included Achondroplasia and several non-ENT pediatric conditions retained only for descriptive context (like the Romanian shorthands “BDA with SDA = 10%” and “BDA with SDA > 10%”, referring to acute diarrheal disease with estimated dehydration of 10% and >10% of body weight, respectively). Non-achondroplasia diagnoses were retained to preserve the consecutive nature of the clinical cohort and to contextualize ENT reporting frequencies in the source population. Importantly, these cases were not used for model-building; all inferential analyses targeting mechanistic interpretation were restricted to the achondroplasia subgroup. The mixed cohort, therefore, provides descriptive background while avoiding inappropriate cross-etiologic inference.

Narrative entries from the “ENT complications” field were mapped (case-insensitive dictionary; Romanian/English variants) to five non-exclusive categories: Otitis media; Adenotonsillar/Apnea, hereafter obstructive sleep apnea (OSA); Audiologic/Eustachian-tube dysfunction (ETD); Nasopharyngeal/upper-respiratory tract (URT); and Extra-ENT (non-ENT comorbidities recorded in the same field). Because categories are non-exclusive, percentages are not additive. Ambiguous expressions were coded conservatively (not positive unless explicit).

In a secondary pass, narrative strings were parsed for OSA qualifiers (severity terms such as “mild/moderate/severe” and type keywords “obstructive/central”); matches were captured as binary flags for descriptive reporting only. Explicit mentions of recurrence and bilaterality were captured as binary indicators to enable the construction of burden scores and, where EPV permitted, inclusion as candidate predictors in multivariable models.

Two summary indices were defined a priori: burden-5 = count of positive ENT categories (0–5) and burden-7 = burden-5 plus recurrence and bilaterality (0–7). These indices operationalize cumulative morbidity at the patient level. Although burden-5 was derived from retrospective categories, its purpose was not causal estimation but pragmatic risk summarization from routinely documented clinical information. Because all five components are available in historical records, retrospective scoring provides a consistent basis for evaluating its discriminative utility.

Two binary outcomes were abstracted from the record: otologic procedures (e.g., tympanostomy tubes) and adenoidectomy/tonsillectomy. Where dates were available, they were used descriptively; modeling used presence/absence.

### 2.3. Statistical Analysis

Source data were entered in Microsoft Excel 2019 (Microsoft Corp., Redmond, WA, USA). A de-identified analysis extract was curated in Excel and analyzed in IBM SPSS Statistics v26 (IBM Corp., Armonk, NY, USA). Core demographic fields included age (years) and gender. Clinical fields comprised a principal diagnosis and a free-text narrative field labeled “ENT complications” (Romanian language source with routine clinical shorthand). Data cleaning addressed obvious typographical variants; all transformations were logged.

Age distribution was assessed using the KS and SW tests. Given marked non-normality, continuous age is summarized as mean ± SD and median [IQR], and categorical variables as *n* (%) with Wilson 95% confidence intervals (CIs) for proportions. Two-tailed Fisher’s exact tests were used for most 2 × 2 contrasts. Age gradients across AC-4 were appraised with an ordinal trend test (logistic regression with the class coded 1–4). A sensitivity contrast used AC-2 (≤5 vs. >5 years).

Year-to-year trend tests were pre-specified for complete years (2023 vs. 2024); observations from January 2025 were summarized descriptively and excluded from inferential comparisons. All ten pairwise 2 × 2 tables among ENT categories were evaluated with Fisher’s exact tests. Multiplicity was controlled using BH-FDR across the ten comparisons; results are reported as odds ratios (ORs) with exact *p*-values and q-values (BH-FDR). In Achondroplasia-only analyses, two pairs were pre-specified, and BH-FDR was applied over those two tests.

Parsimonious logistic models respected EPV constraints. Discrimination was summarized by AUC. Where quasi-separation was detected, Firth-penalized logistic regression was pre-specified. For families of tests, BH-FDR control was applied with primary interpretation based on q. Alongside ORs, absolute risk differences (ARDs) with Newcombe 95% CIs were reported to convey clinical magnitude. Statistical significance was established based on an alpha threshold set at 5%, with *p*-values less than 0.05 indicative of significance, and a confidence interval maintained at 95%.

### 2.4. Ethical Considerations

The study complied with the Declaration of Helsinki and was approved by theEthics Committee of Emergency County Hospital of Craiova (approval number 14274, date 29 March 2022). Given the retrospective design using de-identified data, the requirement for individual informed consent was [waived/obtained, as applicable]. For minors, parental consent was obtained in accordance with institutional policy. When appropriate (typically ≥7 years), child assent was also sought and documented. Because the dataset was fully de-identified for analysis, no re-contact occurred during this retrospective study.

## 3. Results

### 3.1. Cohort Characteristics

#### 3.1.1. Cohort Profile

The study population comprised N = 83 patients. The age distribution was strongly non-normal (Kolmogorov–Smirnov D = 0.166, *p* < 0.001; Shapiro–Wilk W = 0.737, *p* < 0.001), with a mean of 8.53 ± 8.523, a median of 7, an IQR of 8, a 95% CI of 6.67–10.39, and a range of 0–48 years. Shape indices indicated marked right-skew with heavy tails (skewness 2.549; kurtosis 8.062), which supports reporting medians/IQR and the use of non-parametric procedures or Wilson score intervals for proportions. The gender distribution was balanced (female 37, 44.6%; male 46, 55.4%). Regarding case mix, the principal diagnosis was predominantly Achondroplasia (64/83; 77.1%), followed by BDA with SDA = 10% (12; 14.5%), BDA with SDA > 10% (4; 4.8%), disseminated encephalomyelitis (1; 1.2%), and acute interstitial pneumonia (2; 2.4%).

The observation window extended from 1 February 2023 to 31 January 2025. For temporal analyses, 2023 covered February–December (11 months), 2024 covered January–December (12 months), whereas 2025 included January only. To minimize seasonal/length bias, trend tests were pre-specified on complete years (2023 vs. 2024), with 2025 summarized descriptively to avoid over-interpretation. Summary metrics are shown in Table 1.

#### 3.1.2. ENT Phenotypes in the Cohort

Narrative entries from the “ENT complications” field were mapped to five non-exclusive categories: Otitis media, Adenotonsillar/Apnea (first mention; subsequently “OSA”), Audiologic/Eustachian-tube dysfunction (ETD), Nasopharyngeal/upper-respiratory (URT), and Extra-ENT. Percentages are not additive (a patient may contribute to ≥1 category). Confidence intervals are Wilson-type (robust at small/moderate *n*). Category prevalences are summarized in Table 2, with gender-stratified results in Table 3.

Gender-specific distributions are provided in Table 3; no significant differences were observed (all *p* ≥ 0.11), with a borderline signal for the nasopharyngeal/URT category.

#### 3.1.3. Co-Occurrence of ENT Phenotypes

To characterize clustering of ear–nose–throat (ENT) phenotypes within individuals, binary indicators (present/absent) were defined for five non-exclusive categories—Otitis media, Adenotonsillar/Apnea (OSA), Audiologic/Eustachian-tube dysfunction (ETD), Nasopharyngeal/upper-respiratory (URT), and Extra-ENT—and all pairwise combinations among these categories (10 comparisons) were evaluated. Given the moderate sample size and the possibility of sparse cells in 2 × 2 tables, two-tailed Fisher’s exact tests were employed. Multiplicity was addressed using the Benjamini–Hochberg false discovery rate (BH-FDR) across the ten tests, and results are reported as odds ratios (ORs) with exact *p*-values and q-values (BH-FDR). These pairwise analyses are descriptive and do not imply causality; potential confounding by age or gender is explored separately in multivariable models.

The most pronounced co-occurrence linked the otologic pathway with sleep-disordered breathing: Otitis media with OSA (OR = 6.84, *p* = 0.0012, q = 0.0118). The magnitude and direction of this association are physiologically coherent and align with well-described craniofacial influences on Eustachian-tube mechanics and adenotonsillar hypertrophy, which can jointly predispose to middle-ear pathology and obstructive events during sleep. A second signal connected ETD with Extra-ENT involvement (OR = 5.34, *p* = 0.0089, q = 0.0445), suggesting that children manifesting audiologic/tubal dysfunction more often carry non-ENT comorbidities captured in the narrative record; although clinically plausible—via shared susceptibility or healthcare-seeking patterns—this pattern is best regarded as hypothesis-generating. A third pair, Otitis media with ETD, was borderline after multiplicity control (OR = 3.70, *p* = 0.0277, q = 0.0922), consistent with the expected link between inflammatory middle-ear disease and tubal dysfunction yet not surpassing stringent FDR thresholds. Full 2 × 2 results, including ORs and q-values, are reported in Table 4.

After FDR control across the ten planned comparisons, two pairs remained significant (q < 0.05), and one was borderline (q ≈ 0.09). Taken together, the results delineate an otologic axis that concentrates risk across related domains—namely OSA and ETD—thereby providing a rationale for targeted surveillance and timely referral along this clinical corridor. Because pairwise tests cannot adjust for shared drivers (e.g., age strata), the observed clustering is further examined with parsimonious logistic models in Section 3.1.6, where otitis media retains an independent association with ETD.

#### 3.1.4. Age-Stratified Analysis of Key Phenotypes (AC-4)

To appraise potential developmental patterns, prevalence was profiled across the four AC-4 bands (0–2, 3–5, 6–12, ≥13 years) using Wilson intervals and an ordinal trend test (pre-specified). For OSA (Adenotonsillar/Apnea), prevalence rose from early childhood and reached a sustained high level by school age (0–2 years: 0.0% [0/15]; 3–5: 9.5% [2/21]; 6–12: 34.5% [10/29]; ≥13: 33.3% [6/18]; *p*-trend = 0.006). By contrast, the profile for Audiologic/ETD was non-monotonic, with modest fluctuations across bands (0–2: 13.3% [2/15]; 3–5: 19.0% [4/21]; 6–12: 24.1% [7/29]; ≥13: 16.7% [3/18]; *p*-trend = 0.710). Point estimates and Wilson 95% CIs by age band are reported in Table 5.

#### 3.1.5. “ENT Burden” and Probability of Interventions

To capture cumulative morbidity, two summary scores were defined: burden-5 (count of positive ENT categories; range 0–5) and burden-7 (burden-5 plus explicit mentions of recurrence and bilaterality; range 0–7). In parsimonious logistic models adjusted for age and gender, each +1-point increment was associated with higher odds of intervention. For otologic procedures, the odds multiplied by 2.89 per point under burden-5 (OR 2.89; 95% CI 1.37–6.10; *p* = 0.005; AUC 0.83; *n* = 83; events = 9) and by 2.34 under burden-7 (OR 2.34; 95% CI 1.31–4.20; *p* = 0.004; AUC 0.84). For adenoidectomy/tonsillectomy, burden-5 showed a strong association (OR 6.67; 95% CI 2.18–20.43; *p* = 0.001; AUC 0.93; *n* = 83; events = 11), with a smaller but still significant effect for burden-7 (OR 3.89; 95% CI 1.73–8.72; *p* = 0.001; AUC 0.91). Model estimates and performance metrics appear in Table 6.

#### 3.1.6. Targeted Models—Otologic Axis and OSA

Two hypothesis-driven models examined whether the otologic domain independently signals related morbidities after accounting for age and gender.

(i) Audiologic/ETD ~ Otitis media + age + gender. Otitis media remained an independent predictor of audiologic/ETD findings (OR 9.97; 95% CI 1.57–63.21; *p* = 0.015), with AUC 0.744 (*n* = 83; events = 10), indicating moderate discrimination along the otologic–tubal pathway.
(ii) OSA ~ clinical predictors. In a multivariable model including Otitis media, Audiologic/ETD, recurrence, bilaterality, age, and gender, recurrence emerged as the most robust correlate (OR 4.96; 95% CI 1.17–21.05; *p* = 0.030). Otitis media showed a non-significant positive association (OR 3.35; 95% CI 0.68–16.65; *p* = 0.139), while Audiologic/ETD was not informative (OR 1.16; 95% CI 0.19–7.19; *p* = 0.873). Model AUC was 0.775, suggesting acceptable discrimination. Adjusted coefficients and *p*-values are detailed in Table 7.

**Figure 1:**
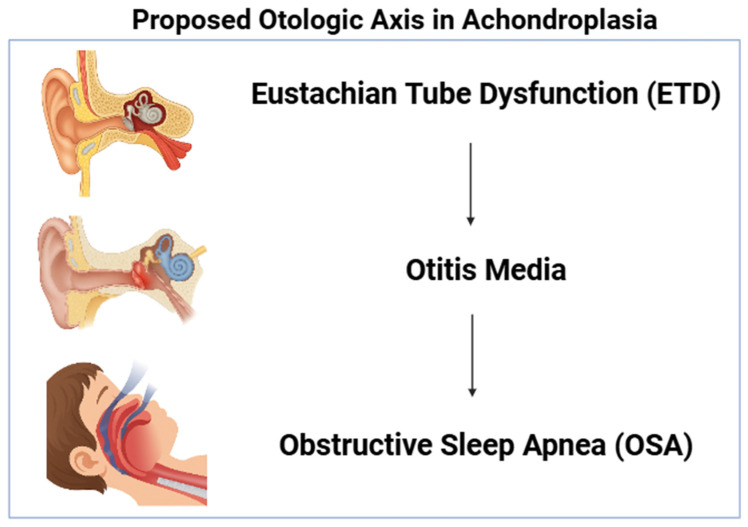
Conceptual schematic of the proposed “otologic axis”. Otitis media functions as the central linkage between ETD and OSA, reflecting the co-occurrence patterns identified in the study. Created with BioRender.com https://app.biorender.com/illustrations/67921226181c1242c9de4622 (accessed on 11 December 2025).

A schematic representation of the proposed “otologic axis” is provided in Figure 1. This illustration summarizes the conceptual pathway observed in the cohort: Eustachian tube dysfunction as an upstream contributor, otitis media as the central and most frequent clinical node, and downstream obstructive sleep apnea reflecting the airway implications of chronic middle-ear and craniofacial dysfunction. The schematic is included to support rapid visualization of the core clinical message and to contextualize the age-structured and co-occurrence patterns reported in the analyses.

### 3.2. Achondroplasia—Focused Analysis

Given the high prevalence of Achondroplasia in the cohort, subsequent analyses focus on Achondroplasia to delineate ENT burden, age structuring, and clinically actionable co-occurrences. Given the study aim, this section prioritizes achondroplasia-specific prevalence, age structuring, and clinically actionable co-occurrences.

#### 3.2.1. Subgroup Profile

The Achondroplasia subgroup comprised 64/83 (77.1%) patients (female 31/64, 48.4%; male 33/64, 51.6%). Age summary was as follows: mean 10.06 ± 9.11, median 8.5, IQR 8.25, range 0–48 years. This distribution indicates substantial spread across developmental stages, which motivated the use of the AC-4 bands for stratified summaries (0–2, 3–5, 6–12, ≥13 years).

#### 3.2.2. ENT Burden and Contrasts Versus Non-Achondroplasia

Given the small non-achondroplasia comparator group (*n* = 19), between-group contrasts should be interpreted as exploratory rather than population-level references.

Within the Achondroplasia subgroup, the dominant phenotypes were Otitis media (51.6%, 95% CI 39.6–63.4) and Adenotonsillar/Apnea—hereafter obstructive sleep apnea (OSA) (28.1%, 95% CI 18.6–40.1). Relative to the non-Achondroplasia remainder (*n* = 19), both conditions showed large absolute risk differences (ARD, Newcombe method) and strong odds-based signals: for Otitis media, ARD +35.8 percentage points (pp) (95% CI +2.0; +57.8), *p* = 0.0077, q (BH-FDR) = 0.0111, OR = 5.68; for OSA, ARD +28.1 pp (95% CI +1.8; +40.1), *p* = 0.0088, q (BH-FDR) = 0.0111, with complete separation in the non-Achondroplasia group (OR → ∞). In contrast, Nasopharyngeal/upper-respiratory (URT) and Extra-ENT categories were substantially less frequent in Achondroplasia, with URT ARD −45.4 pp (95% CI −70.4; −13.5), *p* = 0.00014, q (BH-FDR) = 0.0004, OR = 0.10, and Extra-ENT ARD −47.5 pp (95% CI −72.1; −14.6), *p* = 0.00012, q (BH-FDR) = 0.0004, OR = 0.11. These contrasts—controlled for multiplicity using Benjamini–Hochberg false discovery rate across the five prespecified comparisons—underscore a distinctive ENT burden profile in Achondroplasia, characterized by overrepresentation of otologic disease and OSA alongside relative underrepresentation of URT and extra-ENT problems. Clinical interpretation: the pattern is consistent with craniofacial and airway constraints typical of Achondroplasia, making Otitis media and OSA priority targets for anticipatory screening and management. Comparative prevalences and absolute risk differences (Newcombe) are summarized in Table 8.

#### 3.2.3. Age Dynamics (AC-4) and Sensitivity (Age-Class-2 (AC-2))

Across the pre-specified AC-4 bands, the prevalence of obstructive sleep apnea (OSA) rose from early childhood and peaked at school age, then remained high in adolescence: 0–2 years, 0.0% (0/9); 3–5 years, 18.2% (2/11); 6–12 years, 38.5% (10/26); and ≥13 years, 33.3% (6/18), with an ordinal trend test yielding *p*-trend = 0.0548. In contrast, the age profile for audiologic/Eustachian-tube dysfunction (ETD) showed no consistent gradient: 0–2 years, 0.0% (0/9); 3–5 years, 27.3% (3/11); 6–12 years, 26.9% (7/26); and ≥13 years, 16.7% (3/18), *p*-trend = 0.4866. A sensitivity analysis using a simpler dichotomy (AC-2: ≤5 vs. >5 years) supported concentration of OSA risk beyond age five—10.0% (2/20) versus 36.4% (16/44), Fisher’s exact *p* ≈ 0.03—while differences for ETD were modest at 15.0% (3/20) versus 22.7% (10/44).

#### 3.2.4. Clinical Co-Occurrences in Achondroplasia (Odds Ratios and Absolute Risks)

Evidence of clustering among ENT phenotypes was assessed within the achondroplasia subgroup using two-tailed Fisher’s exact tests with Benjamini–Hochberg control across the two prespecified pairs. Otitis media co-occurred strongly with OSA (odds ratio [OR] 4.97, *p* = 0.0121, q = 0.0121) and with ETD (OR 7.25, *p* = 0.0115, q = 0.0121). Absolute risk metrics corroborated these associations. For OSA, the prevalence was 42.4% (14/33) in those with otitis media versus 12.9% (4/31) without, corresponding to an absolute risk difference (ARD) of +29.5 percentage points (pp) (95% CI −1.6; +54.1), Fisher’s *p* = 0.010. For ETD, prevalence was 33.3% (11/33) with otitis versus 6.5% (2/31) without, ARD +26.9 pp (95% CI −1.0; +48.6), Fisher’s *p* = 0.010. 3.2.5. OSA Severity and Type (Narrative Annotation).

Review of the narrative “ENT complications” field identified explicit labeling in a minority of OSA cases (18/64). Severe OSA was recorded in 1/18 (5.6%), central OSA in 1/18 (5.6%), and obstructive OSA in 2/18 (11.1%), while the remaining episodes lacked severity or type qualifiers. The derivation relied on pattern-matching rules defined a priori (details in Materials and Methods).

#### 3.2.5. Temporal Exploration (Latest Presentation Year, 2023–2025)

When stratified by the latest presentation year within achondroplasia (*n* = 64), point estimates for both OSA and otitis media were lower in the complete 2024 interval than in 2023: OSA, 46.2% (6/13) in 2023 [Feb–Dec] versus 27.9% (12/43) in 2024 [Jan–Dec], Fisher’s *p* = 0.31; otitis media, 69.2% (9/13) versus 55.8% (24/43), *p* = 0.52. The 0.0% (0/8) observation for January 2025 is reported descriptively and was not included in year-to-year testing due to the limited exposure window.

#### 3.2.6. Parsimonious Models in Achondroplasia (Events-per-Variable Respected)

Two minimal logistic models were specified to respect events-per-variable constraints and mitigate overfitting. For OSA (events = 18), the model OSA ~ age + otitis media showed otitis media as an independent correlate (OR 5.03, 95% CI 1.42–17.83, *p* = 0.0123), whereas age was not retained as significant (OR 0.99, 95% CI 0.92–1.07, *p* = 0.835); the area under the curve (AUC) was 0.633. For ETD (events = 13), the model ETD ~ age + otitis media again indicated a dominant otitis signal (OR 9.06, 95% CI 1.63–50.44, *p* = 0.0119) with age non-significant (OR 0.93, 95% CI 0.82–1.06, *p* = 0.305); AUC was 0.750.

## 4. Discussion

This single-center retrospective analysis characterizes ENT morbidity patterns with a pre-specified emphasis on achondroplasia. The cohort displays marked age non-normality and a high proportion of patients with achondroplasia, motivating age-aware summaries and cautious small-sample inference. Against this background, three signals emerge consistently across descriptive and inferential frames: a prominent otologic burden in achondroplasia, a clinically coherent co-occurrence of otologic disease with OSA, and a practical “burden” summary that captures intervention propensity.

Within achondroplasia, otitis media and OSA dominate the ENT profile, whereas nasopharyngeal/URT and extra-ENT entries are comparatively uncommon. ARDs Newcombe complement odds ratios by quantifying clinical magnitude on the probability scale and remain large even after controlling multiplicity via BH-FDR. This pattern is pathophysiologically plausible: craniofacial morphology and Eustachian-tube mechanics in achondroplasia can increase susceptibility to middle-ear disease and sleep-disordered breathing, while URT and heterogeneous extra-ENT issues may contribute less to the core phenotype. The contrast is not merely statistical; it frames the everyday question, “Which ENT problems are most likely in achondroplasia, and by how much?”—to which our results provide numeric answers with uncertainty bounds [8].

Using the four-level age-class schema (AC-4), OSA prevalence rises through early and middle childhood and remains elevated into adolescence, a trajectory compatible with adeno-tonsillar hypertrophy and airway geometry constraints. By contrast, audiologic/ETD shows a non-monotonic profile, suggesting partially independent mechanisms and measurement contexts across ages. A sensitivity contrast with AC-2 (≤5 vs. >5 years) indicates that OSA risk concentrates beyond age five, a clinically intuitive threshold that can guide surveillance schedules. Together, these age-aware analyses argue for stratified monitoring rather than uniform follow-up across childhood.

Pairwise 2 × 2 analyses—reported with exact *p*-values and BH-FDR q-values—show that otitis media co-occurs with OSA and with ETD. The magnitude of association is not trivial: effect sizes are sufficiently large to remain notable after false discovery rate control, and absolute-risk contrasts within achondroplasia underscore practical significance (for example, OSA is several tens of percentage points more frequent when otitis is present). Because pairwise tests cannot disentangle shared drivers, we examined independence in parsimonious logistic models that respect EPV constraints. Within achondroplasia, otitis media retains an independent association with both OSA and ETD, whereas age does not remain significant in these minimal models, likely because age variation is already indirectly reflected in the otologic pathway. This coherence across descriptive, pairwise, and multivariable lenses supports an “otologic axis” as a clinically tractable focus for early identification and referral [9].

An application-facing result is the performance of simple “burden” indices. Both burden-5 (count of positive ENT categories) and burden-7 (burden-5 plus recurrence and bilaterality) discriminate intervention need, with areas under the curve (AUCs) in the acceptable-to-excellent range. Despite its parsimony, burden-5 shows the strongest link to adeno-tonsillar surgery. From a translational perspective, this favors a compact summary readily implementable in clinical notes or electronic forms. Importantly, the indices were conceived a priori and computed from routinely available fields, which enhances reproducibility and facilitates external appraisal. A practical example may clarify how burden-5 could be integrated into real-world triage workflows. In clinical settings where comprehensive audiology or routine PSG cannot be performed for all children with achondroplasia, patients accumulating ≥2–3 burden-5 items—for example, otitis media combined with recurrent URT symptoms or documented ETD—could be prioritized for expedited ENT referral and earlier PSG scheduling. This does not replace clinical judgment, but it provides a structured, reproducible mechanism for identifying higher-risk profiles using variables that are routinely captured in standard documentation. If desired, this triage concept can also be illustrated graphically, such as through a schematic decision pathway or a burden-stratified probability plot.

Temporal summaries by year suggest declining point estimates for OSA and otitis media across complete years (2023 to 2024). Because January 2025 covers a limited time window, those observations were excluded from formal trend tests and are reported descriptively. Small strata and potential shifts in referral patterns advise caution; nonetheless, the direction of change is compatible with improved detection/management or case-mix variation, and warrants continued monitoring in extended series.

Methodological considerations temper the inferences. The retrospective design relies on routine clinical documentation, with ENT complications abstracted from a narrative field. To limit misclassification, mapping used a case-insensitive dictionary across Romanian/English variants and conservative coding of ambiguity. Even so, residual under-capture—particularly for qualifiers such as OSA severity or central versus obstructive type—likely remains. Indeed, explicit labelling of severity/type was sparse; this gap directly motivates structured capture (like polysomnography-based indices and standardized severity/type fields) in subsequent cohorts. Sample size is modest, especially outside the achondroplasia subgroup, and several outcomes are rare. EPV-aware modelling and multiplicity control (BH-FDR) mitigate, but do not eliminate, small-sample risks including separation and imprecise interval estimates. Generalizability may be influenced by single-center context and referral/recording practices; however, methods and codebooks are transparently described to facilitate external replication. In addition, unmeasured socioeconomic, environmental, and healthcare-access factors may have influenced both the presentation and the detection of ENT morbidity in this cohort. Variability in family resources, healthcare-seeking patterns, or regional availability of audiology and polysomnography services could plausibly shape diagnostic pathways and recorded severity. Although such variables were not captured in this dataset and could not be incorporated into EPV-constrained models, their potential contribution should be acknowledged when interpreting the observed patterns.

In settings where polysomnography or comprehensive audiology cannot be performed universally, the otologic axis offers a pragmatic entry point: children with achondroplasia who present with otitis media, especially recurrent/bilateral, likely benefit from lower thresholds for OSA screening and audiologic/ETD assessment. The age-aware structure argues for anticipatory guidance that becomes more proactive beyond age five and remains attentive through the 6–12 year band. The burden-5 summary, because it is simple and discriminative, can support triage to tympanostomy evaluation or adeno-tonsillar surgery pathways without adding documentation burden. These inferences are deliberately framed as risk stratification, not deterministic rules; they should complement, rather than replace, clinician judgement and individualized assessment [10].

First, a standardized, prospective collection of OSA severity (apnea–hypopnea index and related indices) and central versus obstructive classification will close a key information gap and enable calibration of risk scores against graded outcomes. Second, multi-site registries with harmonized dictionaries can expand sample size, improve precision, and test portability across care settings. Third, adding objective measures such as tympanometry, audiometry, and Eustachian-tube function tests would clarify how much of the ETD signal is structural versus infection-driven and whether that distinction modifies OSA risk. Each of these steps is implementable within routine care and directly testable against the burden and co-occurrence structure described here.

In summary, the analysis delineates a concentrated ENT burden in achondroplasia—anchored by otitis media and obstructive sleep apnea—whose age profile and within-patient clustering are clinically coherent and reproducible across analytic lenses. Simple burden indices capture intervention propensity, and otitis media emerges as an actionable marker for targeted OSA and audiologic assessment. While the retrospective design and modest sample counsel interpretive caution, the convergent signals provide a practical scaffold for age-aware surveillance and timely referral. These findings lay the groundwork for prospective, standardized protocols that can both refine risk prediction and improve care pathways in achondroplasia.

The analysis revealed three robust signals within the achondroplasia cohort. First, a concentrated ENT burden was observed, dominated by otitis media and OSA, with large absolute risk differences compared with the non-achondroplasia group, confirmed after BH-FDR correction. In contrast, nasopharyngeal or upper-respiratory tract involvement and extra-ENT entries were substantially less frequent, indicating a specific clustering of morbidity along the otologic–airway axis. Second, an age-structured pattern was evident: OSA prevalence increased steadily through early and middle childhood, peaking in the 6–12-year band and remaining elevated into adolescence, whereas ETD showed a non-monotonic profile without a consistent age gradient. Third, a coherent pattern of clinical co-occurrence emerged, with otitis media associated with both OSA and ETD. These associations persisted in parsimonious logistic models that respected EPV constraints, underscoring their stability and potential causal coherence.

A simple composite index, the five-item “ENT burden” score (burden-5), demonstrated strong discriminative performance for predicting the need for intervention (AUC 0.83–0.93). Despite its simplicity, burden-5 correlated more strongly with adeno-tonsillar surgery than the extended seven-item version (burden-7), supporting its value as a practical and easily implementable clinical summary. Temporal summaries across complete calendar years suggested a downward trend in point estimates between 2023 and 2024, while data from January 2025 were reported descriptively, given the abbreviated observation window.

The main limitations of this work derive from its retrospective single-center design, the reliance on a narrative data field, and the potential under-capture of OSA severity or subtype (central versus obstructive). Between-group comparisons involving the non-achondroplasia subgroup (*n* = 19) are exploratory and may be sensitive to small-sample instability; results should not be generalized beyond descriptive contrast. The burden-5 index was applied retrospectively to narrative documentation; while suitable for exploratory discrimination analyses, its clinical use requires prospective validation and standardized capture of ENT variables. Nevertheless, these limitations were mitigated by the use of non-parametric reporting, multiplicity control through BH-FDR correction, and logistic modeling strategies that minimized the risk of overfitting. The clinical implications are direct: in children with achondroplasia, otitis media may serve as a pragmatic “red flag” for targeted OSA screening and audiologic or Eustachian-tube evaluation, warranting heightened vigilance beyond the age of five years. Looking ahead, standardized collection of OSA severity metrics (including polysomnographic indices and subtype classification), multicenter expansion, and systematic incorporation of objective otologic assessments—such as tympanometry, audiometry, and Eustachian-tube function testing—would refine risk prediction and optimize care pathways for this population.

## 5. Conclusions

This single-center cohort delineates a characteristic ENT burden in achondroplasia, dominated by otitis media and OSA, with large and statistically corrected absolute risk differences compared with non-achondroplasia peers. Age structuring shows that OSA rises through middle childhood and remains elevated into adolescence, whereas audiologic/ETD follows a non-monotonic profile. Within achondroplasia, otitis media clusters with both OSA and ETD and retains an independent association in parsimonious models, indicating an otologic axis that concentrates risk across related ENT domains.

A compact summary index—the five-item ENT burden score—captures intervention need with good discrimination (AUC 0.83–0.93) and, despite its simplicity, aligns more strongly with adeno-tonsillar surgery than the extended variant. These findings support pragmatic workflows in which a history or documentation of otitis media functions as a clinical “red flag” that should prompt targeted screening for OSA (like questionnaire-based triage and polysomnography as indicated) and formal audiologic/ETD assessment, with heightened vigilance after age five.

Interpretation is tempered by the retrospective design, narrative-field abstraction, modest subgroup sizes, and partial-year coverage in 2025; however, risks of multiplicity and overfitting were mitigated through Wilson intervals, Benjamini–Hochberg control, and events-per-variable–constrained modeling. Prospective, multicenter studies with standardized capture of OSA severity and type, alongside objective audiologic and Eustachian-tube measures, are warranted to validate these signals and to refine burden-based risk stratification for people with achondroplasia.

## Figures and Tables

**Table 1:** Cohort characteristics (N = 83): Age-Class-4 (AC-4) bands, numeric age summary, gender, latest presentation year, and principal diagnosis.

Component	Subcomponent	N	
		*n*	%
Age-band distribution (AC-4)	0–2 years	15	18.1
	3–5 years	21	25.3
	6–12 years	29	34.9
	≥13 years	18	21.7
Gender	Female	37	44.6
	Male	46	55.4
Most recent presentation year	2023 (Feb–Dec)	22	26.5
	2024 (Jan–Dec)	53	63.9
	2025 (Jan)	8	9.6
Principal diagnosis	Achondroplasia	64	77.1
	BDA with SDA = 10%	12	14.5
	BDA with SDA > 10%	4	4.8
	Disseminated encephalomyelitis	1	1.2
	Acute interstitial pneumonia	2	2.4

Note: Age distribution is non-normal (KS D = 0.166; SW W = 0.737; both *p* < 0.001). 2023 includes Feb–Dec; 2024 Jan–Dec; 2025 January only. AC-4 band definitions are detailed in Materials and Methods (clinically motivated ENT-risk stages and events-per-variable considerations).

**Table 2:** Prevalence of ENT categories in the cohort (N = 83; 95% CI Wilson).

Component	Positive (*n*)	95% CI (Wilson)	N	
			*n*	%
Otitis media	36	33.2–54.1	83	43.4
Adenotonsillar/Apnea (OSA)	18	14.2–31.7	83	21.7
Audiologic/Eustachian-tube dysfunction (ETD)	16	12.2–29.0	83	19.3
Nasopharyngeal/upper-respiratory (URT)	19	15.2–33.0	83	22.9
Extra-ENT	22	18.2–36.9	83	26.5

**Table 3:** ENT categories by gender (*n*, %) and association test.

ENT Category	Gender		N (*n*/%)	*p* *
	Female (*n* = 37)	Male (*n* = 46)		
Otitis media	13 (35.1%)	23 (50.0%)	36 (43.4%)	0.190
Adenotonsillar/Apnea	6 (16.2%)	12 (26.1%)	18 (21.7%)	0.300
Audiologic/ETD	5 (13.5%)	11 (23.9%)	16 (19.3%)	0.274
Nasopharyngeal/URT	5 (13.5%)	14 (30.4%)	19 (22.9%)	0.113
Extra-ENT	8 (21.6%)	14 (30.4%)	22 (26.5%)	0.456

* Fisher’s exact test.

**Table 4:** Significant/borderline ENT co-occurrences (Fisher’s exact test, BH-FDR across 10 comparisons).

ENT A	ENT B	OR	*p* *	q (BH-FDR)
Otitis media	OSA	6.84	0.0012 ^#^	0.0118
Audiologic/ETD	Extra-ENT	5.34	0.0089	0.0445
Otitis media	Audiologic/ETD	3.70	0.0277	0.0922

* Fisher’s exact test. ^#^ Statistically significant.

**Table 5:** Prevalence by AC-4 classes with 95% CIs (Wilson).

Parameter	Age Class (Years)	*n*/N (%)	95% CI
OSA	0–2	0/15 (0.0)	0.0–20.4
	3–5	2/21 (9.5)	2.7–28.9
	6–12	10/29 (34.5)	19.9–52.7
	≥13	6/18 (33.3)	16.3–56.3
Audiologic/ETD	0–2	2/15 (13.3)	3.7–37.9
	3–5	4/21 (19.0)	7.7–40.0
	6–12	7/29 (24.1)	12.2–42.1
	≥13	3/18 (16.7)	5.8–39.2

**Table 6:** ENT burden → interventions (logistic models).

Outcome	Predictor	OR (95% CI)	*p*	AUC	*n*	Events
Otologic intervention	burden-5	2.89 (1.37–6.10)	0.005 ^#^	0.83	83	9
	burden-7	2.34 (1.31–4.20)	0.004 ^#^	0.84	83	9
Adenoidectomy/tonsillectomy	burden-5	6.67 (2.18–20.43)	0.001 ^#^	0.93	83	11
	burden-7	3.89 (1.73–8.72)	0.001 ^#^	0.91	83	11

^#^ Statistically significant.

**Table 7:** Logistic model for OSA (coefficients and significance).

Predictor	OR	95% CI	*p*
Any recurrence	4.96	1.17–21.05	0.030 ^#^
Otitis media	3.35	0.68–16.65	0.139
Audiologic/ETD	1.16	0.19–7.19	0.873
Age (years)	0.99	0.91–1.08	0.860
Gender (M vs. F)	1.54	0.44–5.35	0.497
Model performance			AUC = 0.775

^#^ Statistically significant.

**Table 8:** Achondroplasia—characterization and contrasts vs. non-Achondroplasia.

Parameter	Value	Achondro		Non-Achondro	OR	*p*	ARD (95% CI)	q
		(*n*/N, %)	95% CI	(*n*/N, %)				
Gender	Female	31/64 (48.4%)	—	6/19 (31.6%)	—	—	—	—
	Male	31/64 (48.4%)	—	6/19 (31.6%)	—	—	—	—
Otitis media	—	33/64 (51.6%)	39.6–63.4	3/19 (15.8%)	5.68	0.0077	+35.8 (+2.0; +57.8)	0.0111
Adenotonsillar/ OSA	—	18/64 (28.1%)	18.6–40.1	0/19 (0.0%)	∞	0.0088	+28.1 (+1.8; +40.1)	0.0111
Audiologic/ETD	—	13/64 (20.3%)	12.3–31.7	3/19 (15.8%)	1.36	1.0000	+4.5 (−25.3; +26.2)	1.0000
Nasopharyngeal/URT	—	8/64 (12.5%)	6.5–22.8	11/19 (57.9%)	0.10	0.00014	−45.4 (−70.4; −13.5)	0.0004
Extra-ENT	—	10/64 (15.6%)	8.7–26.4	12/19 (63.2%)	0.11	0.00012	−47.5 (−72.1; −14.6)	0.0004
OSA by age (AC-4)	0–2	0/9 (0.0%)	0.0–29.9	—	—	—	—	—
	3–5	2/11 (18.2%)	5.1–47.7	—	—	—	—	—
	6–12	10/26 (38.5%)	22.4–57.5	—	—	—	—	—
	≥13	6/18 (33.3%)	16.3–56.3	—	—	—	—	—
Audiologic/ETD by age (AC-4)	0–2	0/9 (0.0%)	0.0–29.9	—	—	—	—	—
	3–5	3/11 (27.3%)	9.7–56.6	—	—	—	—	—
	6–12	7/26 (26.9%)	13.7–46.1	—	—	—	—	—
	≥13	3/18 (16.7%)	5.8–39.2	—	—	—	—	—

Statistically significant; (N = 83; Achondro *n* = 64; Non *n* = 19. 95% CI Wilson for prevalences; 95% CI Newcombe for ARD; Fisher’s exact two-tailed; BH-FDR across the five comparisons).

## Data Availability

The original contributions presented in this study are included in the article. Further inquiries can be directed to the corresponding authors.
